# Dietary Carotenoids Intake and the Risk of Gastric Cancer: A Case—Control Study in Korea

**DOI:** 10.3390/nu10081031

**Published:** 2018-08-07

**Authors:** Ji Hyun Kim, Jeonghee Lee, Il Ju Choi, Young-Il Kim, Oran Kwon, Hyesook Kim, Jeongseon Kim

**Affiliations:** 1 Graduate School of Cancer Science and Policy, National Cancer Center, 323 Ilsan-ro, Ilsandong-gu, Goyang-si, Gyeonggi-do 10408, Korea; 1601009@ncc.re.kr (J.H.K.); jeonghee@ncc.re.kr (J.L.); 2 Center for Gastric Cancer, National Cancer Center Hospital, National Cancer Center, 323 Ilsan-ro, Ilsandong-gu, Goyang-si, Gyeonggi-do 10408, Korea; cij1224@ncc.re.kr (I.J.C.); 11996@ncc.re.kr (Y.-I.K.); 3 Department of Nutritional Science and Food Management, Ewha Womans University, 52, Ewhayeodae-gil, Seodaemun-gu, Seoul 03760, Korea; orank@ewha.ac.kr (O.K.); khs7882@hanmail.net (H.K.)

**Keywords:** carotenoids, lycopene, gastric cancer, *H. pylori*, smoking, case-control study, Korea

## Abstract

Although the incidence of gastric cancer (GC) has declined, it remains the second most common cancer in Korea. As a class of phytochemicals, carotenoids are fat-soluble pigments that are abundant in fruits and vegetables and have health-promoting properties, including cancer prevention effects. This case-control study investigated the effects of total dietary carotenoids, dietary carotenoid subclasses (α-carotene, β-carotene, β-cryptoxanthin, lutein/zeaxanthin, and lycopene), and foods contributing to the dietary intake of each carotenoid on the risk of GC. Four hundred and fifteen cases and 830 controls were recruited from the National Cancer Center Hospital in Korea between March 2011 and December 2014. A significant inverse association between total dietary carotenoids and GC risk was observed among women (odds ratio (OR) 0.56, 95% confidence interval (CI) 0.32–0.99). A higher intake of dietary lycopene was inversely associated with GC risk overall in the subjects (OR 0.60, 95% CI 0.42–0.85, *p* for trend = 0.012), men (OR 0.60, 95% CI 0.39–0.93), and women (OR 0.54, 95% CI 0.30–0.96, *p* for trend = 0.039). This significant association between dietary lycopene intake and GC risk was also observed in the subgroups of *Helicobacter pylori* (*H. pylori*)-positive subjects and those who had ever smoked. Among the major contributing foods of dietary lycopene, consumption of tomatoes and tomato ketchup was inversely associated with GC risk in the overall subjects, men, and women. Based on our findings, a higher intake of dietary lycopene and contributing foods of lycopene (tomatoes and tomato ketchup) may be inversely associated with the risk of GC.

## 1. Introduction

According to the GLOBOCAN estimates reported in 2012, although gastric cancer (GC) incidence is declining, it remains the fifth most common cancer worldwide [1]. GC is the second most common cause of cancer in Korea, as the estimated age-standardized incidence rate of GC was 35.8 per 100,000 persons in 2015 [2]. Therefore, the primary prevention of GC is a major priority in public health.

Several risk factors for GC have been identified, such as *Helicobacter pylori* (*H. pylori*) infection and tobacco smoking, which are classified as International Agency for Research on Cancer (IARC) group 1 carcinogens (carcinogenic to humans) [3,4]. Dietary factors are considered modifiable risk factors that account for approximately 35% of all causes of cancer, and therefore dietary factors related to cancer must be identified [5,6]. 

Differences in the incidence rates of GC subtypes by gender have also been observed [7]. The rate of cardia GC was approximately three times higher in males than in females, whereas the rate of non-cardia GC was two times higher in males than in females [7]. The explanations for the higher rates of GC in males are not known, but might be due to the male predominance of *H. pylori* infection, one of the major risk factors for GC [8]. This phenomenon is found in adults worldwide [8]. The higher incidence rate of GC in males might also be due to the higher consumption of tobacco [9]. However, the predominance of males in the male to female ratio were similar in both smokers and non-smokers; therefore, the higher rates of GC in males might not be completely explained by the smoking history [9].

According to the third expert report from the Continuous Update Project (CUP) published by the World Cancer Research Fund (WCRF) International in 2018, the intake of certain types of foods (e.g., fruits) might be closely related to the risk of GC [10]. Carotenoids are fat-soluble pigments that are highly abundant in fruits and vegetables, and belong to a class of phytochemicals that have health-promoting properties [11,12,13]. Among the more than 40 carotenoids that are derived from a variety of food sources, six types of primary dietary carotenoids are usually detected in human blood plasma (α-carotene, β-carotene, β-cryptoxanthin, lutein, zeaxanthin, and lycopene), suggesting the selective intestinal absorption of these carotenoids [11,12,14].

Several epidemiological studies have been conducted to determine the associations between dietary carotenoids and the risk of GC. However, until now, few studies have been conducted and the results of previous studies are conflicting. Thus, a clear association between dietary carotenoids intake and the risk of GC has not been identified to date. Dietary α-carotene intake was inversely associated with GC risk in some studies [15,16,17,18], whereas no significant associations were observed in other studies [19,20,21]. Dietary β-carotene intake was associated with a reduced risk of GC in some studies [15,16,18,22,23,24], while other studies did not report a significant association [17,19,20,21,25,26]. Studies that analyzed dietary β-cryptoxanthin intake did not identify a significant association with the risk of GC [16,17,18,21]. Dietary lutein/zeaxanthin intake was not significantly associated with the risk of GC [15,16,17,19,20]. Dietary lycopene intake was inversely associated with the risk of GC in one study [17], whereas other studies have found no association [15,16,18,19,20,21,26].

Accordingly, this case-control study aims to investigate the effects of total dietary carotenoids, dietary carotenoid subclasses (α-carotene, β-carotene, β-cryptoxanthin, lutein/zeaxanthin, and lycopene), and the contributing foods of each dietary carotenoid on the risk of GC.

## 2. Materials and Methods

### 2.1. Study Population

The subjects were recruited from the National Cancer Center Hospital in Korea between March 2011 and December 2014. Cases were subjects who had been histologically diagnosed with early GC within the preceding three months at the Center for GC. Early GC was defined as GC restricted to the mucosa or submucosa with or without lymph node metastasis, regardless of the tumor size [27]. Patients in the case group did not have advanced GC, diabetes mellitus, severe systemic/mental disease, or a history of cancer within the past five years, and women who were pregnant or currently breastfeeding were also excluded. Controls were subjects who underwent health-screening examinations at the Center for Cancer Prevention and Detection at the same hospital.

**Figure 1:**
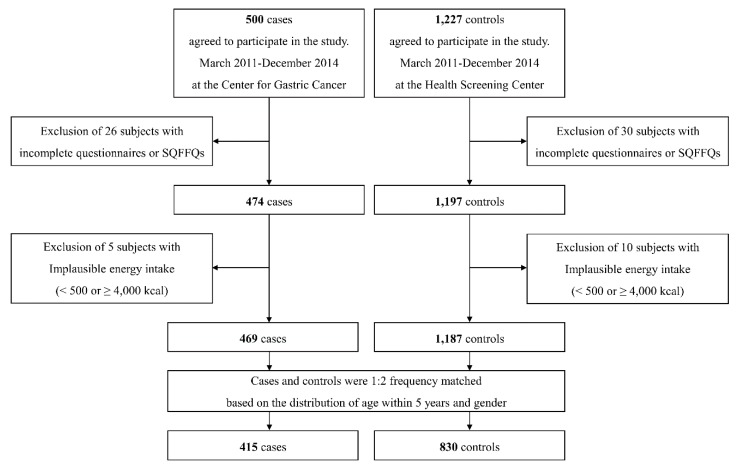
Flow chart of the study subjects. SQFFQ: semi-quantitative food frequency questionnaire.

Among the 1727 subjects (500 cases and 1227 controls) who agreed to participate in the study, 26 cases and 30 controls were excluded due to an incomplete self-administered questionnaire or semi-quantitative food frequency questionnaire (SQFFQ). Of the 1671 subjects remaining, 5 cases and 10 controls were excluded due to the implausibility of a total energy intake of <500 kcal or ≥4000 kcal. Of the 1656 subjects remaining, cases and controls were matched at a ratio of 1:2 by the distribution of age within 5 years and gender. Ultimately, a total of 1245 subjects (415 cases and 830 matched controls; 810 men and 435 women) were selected for this study (Figure 1). Written informed consent was obtained from all participants, and the study protocol was approved by the Institutional Review Board of the National Cancer Center [IRB Number: NCCNCS-11-438].

### 2.2. Data Collection and Management

Participants were asked to complete a self-administered questionnaire that included demographic, lifestyle, and medical history information. Dietary intake was collected from the 106-item SQFFQ, which has been previously reported to be reliable and valid [28]. The study participants in the case group were surveyed with the self-administered questionnaire and SQFFQ by interviewers who were trained beforehand. Participants in the control group were initially asked to complete the survey by themselves, and interviewers asked any questions with missing answers from the self-completed survey during the second round of the survey.

After collecting the dietary information, the amount of each food item consumed was calculated using CAN-PRO 4.0 (Computer Aided Nutritional Analysis Program, The Korean Nutrition Society, Seoul, Korea). The 106 items of the SQFFQ were classified into 663 detailed food items. The overlapping food items were excluded, and 410 food items remained for the analysis. Then, the above food consumption information was merged with the database of carotenoid contents. The carotenoid database used in our study was composed of the United States Department of Agriculture (USDA) carotenoid database [29] and the Food Functional Composition Table provided by the Korea National Academy of Agricultural Science (NAAS) [30]. Additionally, by referring to the recipes from the NAAS Agricultural and Food Integrated Information System, information on seasoned vegetables and kimchi was also included. In total, this database contained 2903 food items. In terms of the carotenoid subclasses, it contained the five main carotenoids, namely, α-carotene, β-carotene, β-cryptoxanthin, lutein/zeaxanthin, and lycopene, and total carotenoids was defined as the sum of the five main carotenoid subclasses.

In terms of matching, except for meat, poultry, seafood, and dairy products, which rarely contain carotenoids (137 food items), the carotenoid database included 98.5% of all food items reported in the SQFFQ. Four food items were excluded due to a lack of information on the carotenoid content. The validity of the SQFFQ for dietary carotenoid intake has been tested using three-day dietary records from 207 people as a gold standard. The crude, energy-adjusted, and energy-adjusted and de-attenuated correlation coefficients for total carotenoids were 0.189, 0.244, and 0.307, respectively. A rapid urease test (Pronto Dry; Medical Instruments Corporation, Solothurn, Switzerland) was conducted to examine the *H. pylori* infection status.

### 2.3. Statistical Analysis

To compare the general characteristics between cases and controls, Student’s *t*-test was used for continuous variables and the chi-square test was used for categorical variables. A contribution analysis was conducted to select the foods contributing to total dietary carotenoids and the carotenoid subclasses. The food items contributing to total dietary carotenoids or carotenoid subclasses that represented up to 90% of the cumulative contribution were selected. All types of dietary carotenoids and their contributing foods were adjusted for total energy intake using the regression residual method [31]. Dietary carotenoids and their contributing foods were categorized by tertiles for the analysis based on the distribution of controls. The lowest tertile of each carotenoid and the foods contributing to dietary intake of each carotenoid were used as references. Odds ratios (ORs) and 95% confidence intervals (CIs) were calculated across the tertiles of dietary carotenoids and their contributing foods using the logistic regression model, after controlling for potential confounding factors. To test for trends, the median values of each tertile category of dietary carotenoids and their contributing foods were used as continuous variables. Model 1 was adjusted for age (as a continuous variable). Model 2 was adjusted for age, total caloric intake (as a continuous variable), a first-degree family history of GC (yes or no), smoking status (current, ex-, or non-smoker), regular exercise status (yes or no), education level (middle school or less, high school, or college or more), occupation (professional and administrative, office and sales/service, labor and agricultural, others, and unemployed), and monthly household income in units of 10,000 won/month (<200, 200–400, or ≥400). Model 3 was adjusted for the *H. pylori* infection status (yes or no) and the variables included in model 2. In the overall subjects, models 1, 2, and 3 were additionally adjusted for gender.

In the stratified analysis according to *H. pylori* infection status, model 1 was adjusted for age and gender. Model 2 was adjusted for age, gender, total caloric intake (as a continuous variable), a first-degree family history of GC (yes or no), smoking status (current, ex-, or non-smoker), regular exercise status (yes or no), education level (middle school or less, high school, or college or more), occupation (professional and administrative, office and sales/service, labor and agricultural, others, and unemployed), and monthly household income in units of 10,000 won/month (<200, 200–400, or ≥400). In the stratified analysis according to smoking status (ever-smoker and non-smoker), model 1 was adjusted for age and gender. Model 2 was adjusted for age, gender, total caloric intake (as a continuous variable), a first-degree family history of GC (yes or no), *H. pylori* infection status (yes or no), regular exercise status (yes or no), education level (middle school or less, high school, or college or more), occupation (professional and administrative, office and sales/service, labor and agricultural, others, and unemployed), and monthly household income in units of 10,000 won/month (<200, 200–400, or ≥400).

All statistical analyses were performed using SAS software (version 9.4, SAS Institute, Cary, NC, USA), and a two-sided *p*-value less than 0.05 was considered statistically significant.

## 3. Results

Table 1 describes the general characteristics of the 415 patients with early GC and 830 controls. Participants in the case group tended to have a higher proportion of *H. pylori* infection (*p* < 0.001) and first-degree family history of GC (*p* = 0.001), a lower percentage of those who had never smoked (*p* < 0.001) and those who exercise regularly (*p* < 0.001), a lower level of education (*p* < 0.001), a different occupational distribution (*p* = 0.001), and a lower level of monthly household income (*p* < 0.001) than those in the control group. Both men and women in the case group had a higher proportion of *H. pylori* infection, a lower proportion of those who never smoked and those who exercise regularly, a lower education level, a different occupational distribution, and a lower level of monthly household income than those in the control group. Additionally, men in the case group had a higher percentage of first-degree family history of GC than the men in the control group.

Table 2 describes the comparison of the consumption of total energy, total dietary carotenoids, and carotenoid subclasses. Subjects in the case group consumed more energy (*p* < 0.001), less total carotenoids (*p* = 0.003), less β-carotene (*p* = 0.018), less β-cryptoxanthin (*p* = 0.007), and less lycopene (*p* < 0.001) than subjects in the control group. Both men and women in the case group consumed less lycopene than the controls. Additionally, men in the case group consumed more energy, and women in the case group consumed less total carotenoids and β-cryptoxanthin compared to controls.

Table 3 shows the ORs and corresponding 95% CIs according to tertiles of total dietary carotenoids and carotenoid subclasses. Among women, higher total carotenoid intake was inversely associated with GC risk (model 2: OR 0.56, 95% CI 0.32–0.99), but the significant association disappeared in model 3, which was additionally adjusted for *H. pylori* infection. A higher lycopene intake was inversely associated with the risk of GC in the overall subjects (model 3: OR 0.60, 95% CI 0.42–0.85, *p* for trend = 0.012), men (model 3: OR 0.60, 95% CI 0.39–0.93), and women (model 2: OR 0.54, 95% CI 0.30–0.96, *p* for trend = 0.039).

Because dietary lycopene intake was significantly associated with GC risk in the overall subjects, a further analysis was performed on lycopene intake. Table 4 shows the ORs and 95% CIs of GC according to tertiles of dietary lycopene intake stratified by *H. pylori* infection status. Among the *H. pylori*-positive subjects, higher lycopene intake was inversely associated with the risk of GC (model 2: OR 0.61, 95% CI 0.42–0.90, *p* for trend = 0.037). When stratified by gender, higher intake of dietary lycopene was associated with a decreased risk of GC among *H. pylori*-positive males (model 2: OR 0.57, 95% CI 0.36–0.91, *p* for trend = 0.043) (Table S1). Table 5 shows the ORs and 95% CIs of GC according to tertiles of dietary lycopene intake stratified by smoking status (ever-smoker and non-smoker). Subjects who currently smoke or previously smoked were combined as ever-smokers. Among ever-smokers, a significantly reduced risk of GC was observed for the subjects who consumed greater amounts of dietary lycopene (model 2: OR 0.39, 95% CI 0.23–0.65, *p* for trend = 0.001). When stratified by gender, higher lycopene intake was inversely associated with the risk of GC among males who has ever smoked (model 2: OR 0.45, 95% CI 0.27–0.73, *p* for trend = 0.005) (Table S2).

Table 6 shows the comparison of the consumption of foods contributing to the dietary intake of lycopene. Compared to controls, cases consumed less tomato in the overall subjects (*p* < 0.001), men (*p* = 0.001), and women (*p* < 0.001); consumed less tomato ketchup in the overall subjects (*p* < 0.001), men (*p* = 0.001), and women (*p* < 0.001); and consumed less watermelon in the overall subjects (*p* = 0.004) and women (*p* = 0.028). Table 7 shows the associations between foods contributing to dietary lycopene intake and the risk of GC. A higher tomato intake was associated with a decreased risk of GC in the overall subjects (model 3: OR 0.59, 95% CI 0.41–0.85, *p* for trend = 0.016), men (model 3: OR 0.58, 95% CI 0.37–0.92, *p* for trend = 0.043), and women (model 2: OR 0.47, 95% CI 0.25–0.87, *p* for trend = 0.010). Higher tomato ketchup intake was associated with a decreased risk of GC in the overall subjects (model 3: OR 0.55, 95% CI 0.38–0.80, *p* for trend = 0.005), men (model 3: OR 0.62, 95% CI 0.39–0.97), and women (model 2: OR 0.47, 95% CI 0.25–0.88, *p* for trend = 0.011).

## 4. Discussion

In our study, an inverse association was observed between higher dietary lycopene intake and the risk of GC in the overall subjects. This significant association remained in the subgroups of gender, *H. pylori*-positive subjects, and those who had ever smoked. The protective effect of dietary lycopene intake on the risk of GC among *H. pylori*–positive subjects and those who had ever smoked was particularly evident in males. Among the contributing foods of dietary lycopene, tomatoes and tomato ketchup exerted protective effects on the risk of GC.

However, previous epidemiological studies of the association between dietary lycopene intake and the risk of GC revealed that lycopene intake is less likely to be associated with GC risk. Among the five case-control studies regarding dietary lycopene intake, a significantly reduced GC risk was observed in only one study conducted in Uruguay (OR 0.37, 95% CI 0.19–0.73) [17]. In other studies, conducted in the US [20], Spain [19], Poland [15], and Italy [16], no significant association was observed. No significant associations between dietary lycopene intake and the risk of GC were observed in three cohort studies conducted in the Netherlands [21], Sweden [18], and in male participants who smoked in Finland [26]. Furthermore, in a meta-analysis that included only studies with validated food frequency questionnaires (FFQs), no statistically significant association was observed between higher dietary lycopene intake with the risk of GC [32]. Another meta-analysis of five case-control studies did not identify any association between higher dietary lycopene intake and the risk of GC [33].

The possible explanation for this trend is that dietary sources and trends of consumption differ among countries and, thus, do not perfectly overlap between studies. Another potential contributing factor is that the studies utilized diverse carotenoid content databases to estimate the consumption of dietary carotenoids, which were linked to various FFQ models. The carotenoid content of each food item was estimated using a different food composition database, such as the USDA database [20,24], the Nutrition Coding Center Nutrient Data System from the University of Minnesota [23], both American and Polish databases [15], Swedish [18,25], Italian [16], North American [17], Spanish [19], Finnish [26], and Dutch databases [21].

In our study, nine lycopene-containing food items were included, in order from the largest contribution to dietary lycopene intake: Watermelons, tomatoes, tomato ketchup, hamburgers, pizza, persimmons, red cabbage, carrots, and pepper powder (data not shown). Three of the nine food items were selected as the primary foods contributing to dietary lycopene intake. Unlike other dietary carotenoid subclasses and total carotenoids, which consist of contributing foods that were different from other studies, foods contributing to dietary lycopene intake were consistent with those from other studies because they included tomatoes and tomato-based products. Tomatoes and tomato-based products accounted for 81.2% of all dietary sources of lycopene in the US, and tomato-based soups and stews accounted for 3.8% [34]. Similarly, in European countries, tomatoes and tomato-based products constituted the major foods contributing to dietary lycopene intake: Tomatoes (25%), canned tomatoes (16%), and pizza (16%) in France; canned tomatoes (23%), tomato soup (17%), and pizza (16%) in Ireland; tomatoes (21%), canned tomatoes (20%), and pizza (15%) in the UK; tomato soup (29%), tomatoes (16%), and pizza (16%) in the Netherlands; and tomatoes (55%) and tomato puree (42%) in Spain [35]. On the other hand, in a previous study conducted in Korea, watermelons, tomatoes, and tomato ketchup contributed to 53.6%, 36.9%, and 5.7% of lycopene intake, respectively [36]. In our study, the major contributors of dietary lycopene intake were the same, but the only difference was the contribution percentage of each food item: 34.56% for watermelons, 32.12% for tomatoes, and 23.58% for tomato ketchup. Although the proportion of each food item that contributed to dietary intake of lycopene differed, the foods contributing to lycopene overlapped, indicating that some country-specific trends in the food items that contribute to lycopene intake might exist.

Among the foods that contribute to dietary lycopene intake, tomatoes and tomato ketchup were significantly associated with the risk of GC. The lycopene content (mg lycopene/100 g) of tomato ketchup is greater than fresh tomatoes [37,38,39]. Lycopene from fresh tomatoes is not readily bioavailable, and thus, by processing tomatoes, the bioavailability of lycopene is increased due to a breakdown of the tissue matrix [40]. This characteristic is due to the structure of the lycopene molecule: Lycopene in raw tomatoes is mainly in the *trans*-isomer form, but, during heat processing, the structure undergoes isomerization to a *cis* form and is thereby more efficiently absorbed [40]. This result is consistent with our finding that the intake of both tomatoes and tomato ketchup were inversely associated with the risk of GC.

Previously, several epidemiological studies on tomato consumption and the risk of GC have been conducted. In a case-control study conducted in the US, an inverse association was observed among only African Americans (OR 0.56, 95% CI 0.34–0.90), but not in Caucasians [41]. In a case-control study conducted in Sweden, a significant inverse association was observed between higher tomato intake (consumed more than 2.9 times per month) during adolescence (OR 0.36, 95% CI 0.23–0.58, *p* for trend < 0.0001) [42]. However, other case-control studies conducted in Spain [43], Sweden [22], Japan [44], and the US [45] did not identify any significant associations. In a cohort study conducted in the Netherlands, a borderline positive association was found between tomato consumption and non-cardia GC risk (relative risk (RR) per 25 g/day increase in the amount of tomatoes consumed: 1.13, 95% CI 1.00–1.28) [46]. However, when a meta-analysis was conducted with those seven studies listed above that have validated FFQs, tomato consumption was significantly associated with a decreased risk of GC (OR 0.73, 95% CI 0.60–0.90), with moderate heterogeneity (*I*^2^ = 47.92%) [32]. This finding is consistent with our results because we also observed the protective effects of tomatoes and tomato ketchup on the risk of GC.

In our study, lycopene was inversely associated with the risk of GC, and the association remained significant in *H. pylori*-positive subjects and those who had ever smoked. There is a possible explanation of those risk factors and the effect of lycopene on GC prevention. Smoking, inflammation, and *H. pylori* infection may increase oxidative stress in the gastrointestinal tract, which leads to DNA damage, extracellular signal-regulated kinase (ERK) activation and p53 induction, decreased activities of antioxidant enzymes (glutathione, GSH; glutathione-S-transferase, GST; and glutathione peroxidase, GPx), and impaired immune function [46]. Lycopene might scavenge reactive oxygen species (ROS) and stimulate antioxidant enzyme activities, which protect gastric mucosa from oxidative stress-induced ERK activation, p53 induction, cell cycle disturbances, and impaired immune function, thereby, preventing gastric carcinogenesis [47]. Lycopene might scavenge ROS and stimulate antioxidant enzyme activities, which protect the gastric mucosa from oxidative stress-induced ERK activation, p53 induction, cell cycle disturbances, and impaired immune function, thereby, preventing gastric carcinogenesis [47].

However, regarding contributing foods, it would be presumptuous to assume that certain foods are representative of a specific nutrient, and to conclude that a food item protects against GC because of the specific nutrient. Foods contributing to dietary carotenoid intake are mostly fruits and vegetables, but they are also good sources of bioactive phytochemicals [48]. In a Spanish case-control study, a higher intake of dietary flavonoids, particularly kaempferol, exerted a protective effect on the risk of GC, while no significant association was observed for carotenoids [19]. Tomatoes are rich in carotenoids, but they also contain lower concentrations of polyphenols, such as hydroxycinnamic acids, flavanones, flavonols, anthocyanins, and flavonol glycosides [49]. In addition, tomatoes are a relatively rich source of vitamin C [32]. In vivo and in vitro studies have suggested that bioactive compounds in tomatoes may work additively or synergistically to reduce the growth of cancer cells [12]. However, since both higher dietary lycopene consumption and higher tomato intake were inversely associated with the risk of GC in our study, we may still suggest a possible effect of dietary lycopene intake by consuming tomatoes and tomato ketchup, which contributed to 55.7% of the total lycopene intake. 

Our study has certain strengths: (1) A comprehensive and validated 106-item SQFFQ was used; (2) the study participants in the case group were surveyed by trained interviewers. Subjects in the control group were initially asked to complete the survey by themselves, and trained interviewers asked any questions with missing answers in a second session. Therefore, the quality of data was improved; (3) the carotenoid database included the contents of kimchi and seasoned vegetables, and covered 98.5% of all items reported in the SQFFQ; (4) information on the prevalence of *H. pylori* infection and smoking status, which are known risk factors for GC according to the IARC, were available [3,4]; and (5) to our knowledge, this is the first study conducted in Korea to investigate the association between dietary carotenoids and GC risk.

Several limitations should also be mentioned: (1) In this hospital-based, case-control study, selection bias might have occurred because the controls were those who had participated in the health screening. Subjects who chose to undergo screening may have had healthier lifestyles and dietary habits (e.g., greater consumption of fruits and vegetables) than individuals who did not choose to undergo screening; thus, the controls might be less representative of the general population; (2) our findings might be prone to recall bias because subjects were required to report their dietary intake for the past 12 months, which is a relatively long period. Additionally, when the SQFFQ was assessed, cases had already received a confirmed diagnosis; thus, the recall ability may have differed between cases and controls. However, in our study, cases were only patients who were diagnosed with early GC. Thus, compared to advanced GC cases, the influence of dietary changes on GC symptoms will be negligible. Moreover, cases with other health factors that might have affected their diet (subjects with diabetes mellitus, a severe systematic/mental disease, a history of cancer within five years, and women who were pregnant or currently breastfeeding) were excluded. Therefore, the recall bias in this study might be minimal; and (3) the sample size was relatively small in our study; in particular, few *H. pylori*-negative subjects and subjects with cardia GC were included. However, the higher percentage of cases with non-cardia GC among the overall GC incidence is a unique trend in Asian countries, including Korea [7]; nevertheless, a larger sample size is needed to increase the statistical power.

## 5. Conclusions

Based on our findings, higher dietary lycopene intake might be inversely associated with the risk of GC in the overall subjects. The association remained significant in the subgroups of gender, *H. pylori*-positive subjects, and those who had ever smoked. Foods contributing to dietary lycopene that exerted protective effects on the risk of GC were tomatoes and tomato ketchup. Further studies with larger sample sizes, including sufficient numbers of *H. pylori*-negative subjects and patients with cardia GC, are needed. 

## Figures and Tables

**Table 1:** General characteristics of the study subjects ^a^.

	Total (*n* = 1245)			Men (*n* = 810)			Women (*n* = 435)		
	Controls (*n* = 830)	Cases (*n* = 415)	*p* ^b^	Controls (*n* = 540)	Cases (*n* = 270)	*p* ^b^	Controls (*n* = 290)	Cases (*n* = 145)	*p* ^b^
**Age (years)**	53.7 ± 9.0	53.8 ± 9.3	0.892	54.8 ± 8.4	54.9 ± 8.7	0.905	51.6 ± 9.8	51.7 ± 10.0	0.942
<50	285 (34.3)	139 (33.5)	0.816	153 (28.3)	77 (28.5)	>0.999	132 (45.5)	62 (42.8)	0.658
≥50	545 (65.7)	276 (66.5)		387 (71.7)	193 (71.5)		158 (54.5)	83 (57.2)	
**Male, *n* (%)**	540 (65.1)	270 (65.1)	>0.999						
**BMI (kg/m^2^)**	23.9 ± 2.9	23.9 ±3.0	0.627	24.4 ± 2.7	24.2 ± 3.0	0.390	23.1 ± 3.1	23.2 ± 3.0	0.533
<23	314 (37.8)	159 (38.3)	0.975	161 (29.8)	91 (33.7)	0.509	153 (52.8)	68 (46.9)	
23–25	249 (30.0)	122 (29.4)		170 (31.5)	78 (28.9)		79 (27.2)	44 (30.3)	
≥25	266 (32.1)	133 (32.1)		209 (38.7)	101 (37.4)		57 (19.7)	32 (22.1)	
***H. pylori* infection**									
Positive	486 (58.6)	382 (92.1)	<0.001	333 (61.7)	252 (93.3)	<0.001	153 (52.8)	130 (89.7)	<0.001
Negative	320 (38.6)	33 (8.0)		187 (34.6)	18 (6.7)		133 (45.9)	15 (10.3)	
**First-degree family history of GC**									
Yes	103 (12.4)	82 (19.8)	0.001	74 (13.7)	60 (22.2)	0.003	29 (10.0)	22 (15.2)	0.155
No	725 (87.4)	332 (80.0)		464 (85.9)	209 (77.4)		261 (90.0)	123 (84.8)	
**Smoking status, *n* (%)**									
Current-smoker	162 (19.5)	128 (30.8)	<0.001	157 (29.1)	121 (44.8)	<0.001	5 (1.7)	7 (4.8)	0.021
Ex-smoker	284 (34.2)	119 (28.7)		277 (51.3)	110 (40.7)		7 (2.4)	9 (6.2)	
Non-smoker	384 (46.3)	167 (40.2)		106 (19.6)	39 (14.4)		278 (95.9)	128 (88.3)	
**Alcohol intake**									
Current-drinker	534 (64.3)	254 (61.2)	0.243	404 (74.8)	193 (71.5)	0.282	130 (44.8)	61 (42.1)	0.819
Ex-drinker	60 (7.2)	41 (9.9)		47 (8.7)	33 (12.2)		13 (4.5)	8 (5.5)	
Non-drinker	236 (28.4)	119 (28.7)		89 (16.5)	44 (16.3)		147 (50.7)	75 (51.7)	
**Regular exercise**									
Yes	466 (56.1)	147 (35.4)	<0.001	303 (56.1)	109 (40.4)	<0.001	163 (56.2)	38 (26.2)	<0.001
No	361 (43.5)	268 (64.6)		234 (43.3)	161 (59.6)		127 (43.8)	107 (73.8)	
**Education, *n* (%)**									
Middle school or less	119 (14.3)	142 (34.2)	<0.001	71 (13.2)	91 (33.7)	<0.001	48 (16.6)	51 (35.2)	<0.001
High school	253 (30.5)	174 (41.9)		140 (25.9)	112 (41.5)		113 (39.0)	62 (42.8)	
College or more	426 (51.3)	97 (23.4)		301 (55.7)	66 (24.4)		125 (43.1)	31 (21.4)	
**Marital status, *n* (%)**									
Married	716 (86.3)	361 (87.0)	0.674	478 (88.5)	243 (90.0)	0.553	238 (82.1)	118 (81.4)	>0.999
Others	113 (13.6)	52 (12.5)		61 (11.3)	26 (9.6)		52 (17.9)	26 (17.9)	
**Occupation, *n* (%)**									
Professional, administrative	156 (18.8)	70 (16.9)	0.001	117 (21.7)	59 (21.9)	0.010	39 (13.5)	11 (7.6)	0.002
Office, sales and service	266 (32.1)	122 (29.4)		203 (37.6)	81 (30.0)		63 (21.7)	41 (28.3)	
Laborer, agricultural	128 (15.4)	104 (25.1)		111 (20.6)	83 (30.7)		17 (5.9)	21 (14.5)	
Others, unemployed	277 (33.4)	117 (28.2)		106 (19.6)	46 (17.0)		171 (59.0)	71 (49.0)	
**Monthly household income,** **10,000 won/month, *n* (%)**									
<200	149 (18.0)	133 (32.1)	<0.001	85 (15.7)	85 (31.5)	<0.001	64 (22.1)	48 (33.1)	0.016
200–400	341 (41.1)	148 (35.7)		232 (43.0)	106 (39.3)		109 (37.6)	42 (29.0)	
>400	273 (32.9)	96 (23.1)		168 (31.1)	55 (20.4)		105 (36.2)	41 (28.3)	
**Histological subtype of GC** **(Lauren’s classification)**									
Intestinal	-	158 (38.1)	-	-	132 (48.9)	-	-	26 (17.9)	-
Diffuse	-	164 (39.5)		-	77 (28.5)		-	87 (60.0)	
Mixed	-	59 (14.2)		-	40 (14.8)		-	19 (13.1)	
Indeterminate	-	4 (1.0)		-	3 (1.1)		-	1 (0.7)	

Missing data are included in the total %. ^a^ Values are presented as the means ± standard deviations (SD) or *n* (%); ^b^ *p*-values for continuous variables and categorical variables were calculated using Student’s *t*-test and the chi-square test, respectively. BMI: body mass index; GC: gastric cancer.

**Table 2:** Comparison of the consumption of total energy, total dietary carotenoids, and carotenoid subclasses ^a^.

	Total (*n* = 1245)			Men (*n* = 810)			Women (*n* = 435)		
	Controls (*n* = 830)	Cases (*n* = 415)	*p* ^b^	Controls (*n* = 540)	Cases (*n* = 270)	*p* ^b^	Controls (*n* = 290)	Cases (*n* = 145)	*p* ^b^
**Total energy intake (kcal)**	1713.6 ± 545.5	1924.1 ± 612.9	<0.001	1760.6 ± 541.5	2038.5 ± 634.8	<0.001	1626.0 ± 543.1	1711.1 ± 507.0	0.116
**Total carotenoid intake (μg/day)**									
Total carotenoids	12,121.5 ± 7762.4	10,799.2 ± 6987.1	0.003	10,785.7 ± 6632.5	9990.7 ± 6294.5	0.102	14,608.7 ± 9014.1	12,304.7 ± 7927.0	0.009
α-Carotene	947.5 ± 913.8	925.3 ± 966.0	0.692	839.8 ± 805.4	833.1 ± 861.7	0.913	1148.0 ± 1059.9	1096.9 ± 1118.1	0.642
β-Carotene	5075.9 ± 3276.2	4632.6 ± 3009.4	0.018	4529.7 ± 2682.8	4226.4 ± 2568.0	0.124	6093.1 ± 3971.2	5388.9 ± 3582.4	0.073
β-Cryptoxanthin	393.7 ± 438.8	330.5 ± 364.6	0.007	310.0 ± 269.7	314.3 ± 390.1	0.872	549.6 ± 615.7	360.6 ± 310.5	<0.001
Lutein/Zeaxanthin	3531.7 ± 2584.6	3455.2 ± 2764.4	0.631	3256.6 ± 2200.2	3268.1 ± 2726.8	0.952	4044.0 ± 3119.2	3803.7 ± 2809.2	0.435
Lycopene	2218.0 ± 3847.2	1439.0 ± 2135.7	<0.001	1869.8 ± 3433.0	1312.1 ± 2110.4	0.005	2866.4 ± 4452.9	1675.3 ± 2169.5	<0.001

^a^ Adjusted for total energy intake using the residuals method; ^b^ *p*-values were calculated using Student’s *t*-test.

**Table 3:** Odds ratios (ORs) and 95% confidence intervals (CIs) of gastric cancer (GC) according to the tertiles of total dietary carotenoids and carotenoid subclasses ^a^.

	Median Intake (μg/day)	No. of Controls/Cases	Model 1		Model 2		Model 3	
			OR	(95% CI)	OR	(95% CI)	OR	(95% CI)
**Total Carotenoids**								
Total (*n* = 1245)								
T1	6064.75	276/162	1.00		1.00		1.00	
T2	10,171.44	277/146	0.88	(0.67–1.17)	0.98	(0.71–1.34)	1.01	(0.72–1.42)
T3	17,946.94	277/107	0.64	(0.47–0.86)	0.75	(0.53–1.06)	0.79	(0.55–1.15)
*p* for trend ^b^			0.003		0.082		0.185	
Men (*n* = 810)								
T1	5603.87	180/106	1.00		1.00		1.00	
T2	8921.20	180/88	0.83	(0.58–1.17)	0.95	(0.63–1.43)	1.09	(0.70–1.68)
T3	15,850.62	180/76	0.71	(0.49–1.02)	0.78	(0.51–1.20)	0.84	(0.54–1.33)
*p* for trend ^b^			0.073		0.236		0.386	
Women (*n* = 435)								
T1	7151.14	96/74	1.00		1.00		1.00	
T2	12,377.35	97/38	0.50	(0.31–0.81)	0.51	(0.30–0.88)	0.51	(0.29–0.91)
T3	21,678.14	97/33	0.43	(0.26–0.71)	0.56	(0.32–0.99)	0.65	(0.35–1.18)
*p* for trend ^b^			0.001		0.051		0.169	
**α-Carotene**								
Total (*n* = 1245)								
T1	321.92	276/151	1.00		1.00		1.00	
T2	665.17	277/136	0.90	(0.67–1.19)	0.99	(0.71–1.36)	0.94	(0.67–1.33)
T3	1527.25	277/128	0.84	(0.62–1.12)	0.99	(0.71–1.38)	1.00	(0.70–1.41)
*p* for trend ^b^			0.269		0.969		0.957	
Men (*n* = 810)								
T1	291.35	180/100	1.00		1.00		1.00	
T2	587.52	180/78	0.78	(0.54–1.12)	0.95	(0.63–1.44)	0.95	(0.61–1.48)
T3	1404.67	180/92	0.92	(0.65–1.31)	0.98	(0.65–1.48)	0.95	(0.61–1.47)
*p* for trend ^b^			0.879		0.972		0.839	
Women (*n* = 435)								
T1	427.91	96/52	1.00		1.00		1.00	
T2	853.57	97/42	0.80	(0.49–1.31)	0.84	(0.48–1.48)	0.83	(0.46–1.50)
T3	1704.44	97/51	0.97	(0.60–1.57)	1.48	(0.85–2.58)	1.49	(0.83–2.70)
*p* for trend ^b^			0.961		0.113		0.125	
**β-Carotene**								
Total (*n* = 1245)								
T1	2574.39	276/162	1.00		1.00		1.00	
T2	4339.20	277/134	0.82	(0.61–1.08)	0.81	(0.58–1.11)	0.84	(0.60–1.18)
T3	7353.32	277/119	0.71	(0.53–0.96)	0.77	(0.55–1.08)	0.85	(0.59–1.22)
*p* for trend ^b^			0.030		0.158		0.426	
Men (*n* = 810)								
T1	2447.55	180/100	1.00		1.00		1.00	
T2	3791.98	180/93	0.93	(0.65–1.32)	1.00	(0.67–1.50)	1.10	(0.72–1.70)
T3	6504.49	180/77	0.76	(0.53–1.10)	0.76	(0.49–1.17)	0.89	(0.56–1.41)
*p* for trend ^b^			0.136		0.179		0.535	
Women (*n* = 435)								
T1	3178.03	96/68	1.00		1.00		1.00	
T2	5104.57	97/35	0.50	(0.31–0.83)	0.52	(0.30–0.92)	0.53	(0.29–0.97)
T3	8683.53	97/42	0.60	(0.37–0.97)	0.73	(0.42–1.25)	0.79	(0.44–1.41)
*p* for trend ^b^			0.062		0.351		0.551	
**β-Cryptoxanthin**								
Total (*n* = 1245)								
T1	128.90	276/170	1.00		1.00		1.00	
T2	270.81	277/136	0.79	(0.60–1.05)	0.90	(0.66–1.24)	0.94	(0.67–1.32)
T3	594.01	277/109	0.62	(0.46–0.84)	0.79	(0.56–1.10)	0.77	(0.54–1.10)
*p* for trend ^b^			0.003		0.172		0.142	
Men (*n* = 810)								
T1	116.88	180/104	1.00		1.00		1.00	
T2	229.41	180/86	0.83	(0.58–1.18)	0.86	(0.57–1.30)	0.96	(0.62–1.49)
T3	483.12	180/80	0.77	(0.54–1.10)	0.85	(0.56–1.29)	0.92	(0.59–1.43)
*p* for trend ^b^			0.180		0.502		0.717	
Women (*n* = 435)								
T1	180.27	96/68	1.00		1.00		1.00	
T2	369.16	97/46	0.67	(0.42–1.07)	0.92	(0.54–1.57)	0.84	(0.47–1.48)
T3	851.83	97/31	0.45	(0.27–0.75)	0.60	(0.34–1.07)	0.55	(0.30–1.02)
*p* for trend ^b^			0.003		0.076		0.054	
**Lutein/Zeaxanthin**								
Total (*n* = 1245)								
T1	1716.04	276/152	1.00		1.00		1.00	
T2	2832.71	277/135	0.88	(0.66–1.17)	0.93	(0.67–1.28)	1.00	(0.71–1.41)
T3	5188.69	277/128	0.83	(0.62–1.12)	0.87	(0.63–1.22)	0.91	(0.64–1.30)
*p* for trend ^b^			0.245		0.440		0.575	
Men (*n* = 810)								
T1	1676.82	180/91	1.00		1.00		1.00	
T2	2691.75	180/89	0.98	(0.68–1.40)	1.18	(0.78–1.79)	1.41	(0.91–2.20)
T3	4679.81	180/90	0.99	(0.69–1.41)	1.03	(0.68–1.58)	1.13	(0.72–1.76)
*p* for trend ^b^			0.954		0.996		0.788	
Women (*n* = 435)								
T1	1894.17	96/61	1.00		1.00		1.00	
T2	3268.31	97/39	0.63	(0.38–1.03)	0.60	(0.35–1.05)	0.64	(0.36–1.15)
T3	5678.59	97/45	0.71	(0.44–1.17)	0.83	(0.48–1.45)	0.82	(0.45–1.47)
*p* for trend ^b^			0.240		0.636		0.602	
**Lycopene**								
Total (*n* = 1245)								
T1	327.68	276/209	1.00		1.00		1.00	
T2	1105.49	277/112	0.53	(0.40–0.70)	0.67	(0.49–0.92)	0.67	(0.48–0.94)
T3	3666.52	277/94	0.44	(0.33–0.59)	0.57	(0.41–0.80)	0.60	(0.42–0.85)
*p* for trend ^b^			<0.001		0.003		0.012	
Men (*n* = 810)								
T1	268.48	180/138	1.00		1.00		1.00	
T2	934.05	180/65	0.47	(0.33–0.68)	0.51	(0.34–0.78)	0.55	(0.35–0.86)
T3	2963.59	180/67	0.49	(0.34–0.69)	0.60	(0.40–0.91)	0.60	(0.39–0.93)
*p* for trend ^b^			0.001		0.056		0.062	
Women (*n* = 435)								
T1	442.87	96/69	1.00		1.00		1.00	
T2	1528.34	97/46	0.66	(0.41–1.05)	0.77	(0.45–1.31)	0.81	(0.46–1.42)
T3	4843.59	97/30	0.43	(0.26–0.72)	0.54	(0.30–0.96)	0.60	(0.32–1.11)
*p* for trend ^b^			0.002		0.039		0.113	

^a^ Total dietary carotenoids and carotenoid subclasses were categorized into tertiles according to the distribution of the control groups: Total carotenoids for overall subjects (T1: 7932.26, T2: 7932.26–13,062.36, and T3: ≥13,062.36), men (T1: <7393.71, T2: 7393.71–11,605.58, and T3: ≥11,605.58), and women (T1: <10,177.56, T2: 10,177.56–16,039.47, and T3: ≥16,039.47); α-carotene for overall subjects (T1: <480.37, T2: 480.37–956.11, and T3: ≥956.11), men (T1: <435.49, T2: 435.49–799.48, and T3: ≥799.48), and women (T1: <587.17, T2: 587.17–1160.14, and T3: ≥1160.14); β-carotene for overall subjects (T1: <3370.16, T2: 3370.16–5327.21, and T3: ≥5327.21), men (T1: <3165.51, T2: 3165.51–4843.61, and T3: ≥4843.61), and women (T1: <4238.05, T2: 4238.05–6436.30, and T3: ≥6436.30); β-cryptoxanthin for overall subjects (T1: <190.36, T2: 190.36–381.30, and T3: ≥381.30), men (T1: <166.94, T2: 166.94–322.32, and T3: ≥322.32), and women (T1: <254.69, T2: 254.69–510.98, and T3: ≥510.98); lutein/zeaxanthin for overall subjects (T1: <2242.79, T2: 2242.79–3,707.97, and T3: ≥3707.97), men (T1: <2122.45, T2: 2122.45–3379.04, and T3: ≥3379.04), and women (T1: <2507.60, T2: 2507.60–4218.61, and T3: ≥4218.61); and lycopene for overall subjects (T1: <683.61, T2: 683.61–1881.13, and T3: ≥1881.13), men (T1: <574.69, T2: 574.69–1590.15, and T3: ≥1590.15), and women (T1: <898.55, T2: 898.55–2572.63, and T3: ≥2572.63). ^b^ To test for a trend across tertiles, the median intake for each tertile category was used as a continuous variable. Model 1: Adjusted for age; Model 2: Adjusted for age, total caloric intake, family history of GC, smoking status, regular exercise, education level, occupation, and monthly household income; Model 3: Additionally adjusted for *H. pylori* infection. In the overall subjects, models 1, 2, and 3 were additionally adjusted for gender.

**Table 4:** ORs and 95% CIs of GC according to tertiles of dietary lycopene intake stratified by *H. pylori* infection status ^a^.

	Median Intake (μg/day)	*H. pylori*-Positive (*n* = 868)					*H. pylori*-Negative (*n* = 353)				
		No. of Controls/Cases	Model 1		Model 2		No. of Controls/Cases	Model 1		Model 2	
			OR	(95% CI)	OR	(95% CI)		OR	(95% CI)	OR	(95% CI)
**Lycopene**											
Total (*n* = 1221)											
T1	327.68	169/194	1.00		1.00		100/15	1.00		1.00	
T2	1105.49	166/100	0.52	(0.37–0.72)	0.60	(0.42–0.86)	102/12	0.75	(0.33–1.69)	1.23	(0.48–3.18)
T3	3666.52	151/88	0.49	(0.35–0.69)	0.61	(0.42–0.90)	118/6	0.30	(0.11–0.81)	0.50	(0.16–1.54)
*p* for trend ^b^			<0.001		0.037			0.017		0.168	

^a^ Dietary lycopene intake was categorized into tertiles according to the distribution of the control group among overall subjects (T1: <683.61, T2: 683.61–1881.13, and T3: ≥1881.13). ^b^ To test for trend across tertiles, the median intake for each tertile category was used as a continuous variable. Model 1: Adjusted for age and gender; Model 2: Adjusted for age, gender, total caloric intake, family history of GC, smoking status, regular exercise, education level, occupation, and monthly household income.

**Table 5:** ORs and 95% CIs of GC according to tertiles of dietary lycopene intake stratified by smoking status ^a^.

	Median Intake (μg/day)	Ever-Smoker (*n* = 693) ^b^					Non-Smoker (*n* = 551)				
		No. of Controls/Cases	Model 1		Model 2		No. of Controls/Cases	Model 1		Model 2	
			OR	(95% CI)	OR	(95% CI)		OR	(95% CI)	OR	(95% CI)
**Lycopene**											
Total (*n* = 1244)											
T1	327.68	166/138	1.00		1.00		110/70	1.00		1.00	
T2	1105.49	152/63	0.49	(0.34–0.71)	0.49	(0.31–0.77)	125/49	0.61	(0.39–0.96)	0.88	(0.52–1.48)
T3	3666.52	128/46	0.40	(0.26–0.60)	0.39	(0.23–0.65)	149/48	0.49	(0.32–0.77)	0.75	(0.45–1.27)
*p* for trend ^c^			<0.001		0.001			0.006		0.307	

^a^ Dietary lycopene intake was categorized into tertiles according to the distribution of the control group among overall subjects (T1: <683.61, T2: 683.61–1881.13, and T3: ≥1881.13). ^b^ Subjects who currently smoke or previously smoked were combined as ever-smokers. ^c^ To test for trend across tertiles, the median intake for each tertile category was used as a continuous variable. Model 1: Adjusted for age and gender; Model 2: Adjusted for age, gender, total caloric intake, family history of GC, *H. pylori* infection, regular exercise, education level, occupation, and monthly household income.

**Table 6:** Comparison of the consumption of lycopene contributing foods ^a^.

Food Consumption (g/day)		Total (*n* = 1245)			Men (*n* = 810)			Women (*n* = 435)		
	Cumulative (%) ^b^	Controls (*n* = 830)	Cases (*n* = 415)	*p* ^c^	Controls (*n* = 540)	Cases (*n* = 270)	*p* ^c^	Controls (*n* = 290)	Cases (*n* = 145)	*p* ^c^
Watermelon	34.56	21.02 ± 69.39	13.45 ± 21.73	0.004	15.25 ± 36.47	11.27 ± 21.19	0.050	31.75 ± 105.62	17.50 ± 22.22	0.028
Tomato	66.68	32.61 ± 90.50	14.71 ± 31.94	<0.001	27.01 ± 91.31	12.68 ± 32.60	0.001	43.05 ± 88.19	18.50 ± 30.43	<0.001
Tomato ketchup	90.26	5.64 ± 15.31	2.62 ± 5.67	<0.001	4.71 ± 15.51	2.28 ± 5.79	0.001	7.38 ± 14.81	3.25 ± 5.40	<0.001

^a^ Adjusted for total energy intake using the residuals method. ^b^ Food items contributing to dietary lycopene intake that represented up to 90% of the cumulative contribution were selected. ^c^ *p*-values were calculated using Student’s *t*-test.

**Table 7:** ORs and 95% CIs of GC according to tertiles of the consumption of lycopene contributing foods.

	Range (g/day)	Median Intake (g/day)	No. of Controls/Cases	Model 1		Model 2		Model 3	
				OR	(95% CI)	OR	(95% CI)	OR	(95% CI)
**Watermelon**									
Total (*n* = 1245)									
T1	<3.91	1.47	276/188	1.00		1.00		1.00	
T2	3.91–13.59	7.23	277/116	0.61	(0.46–0.81)	0.75	(0.54–1.04)	0.73	(0.52–1.03)
T3	≥13.59	29.32	277/111	0.58	(0.43–0.77)	0.77	(0.55–1.07)	0.71	(0.50–1.02)
*p* for trend ^a^				0.002		0.233		0.130	
Men (*n* = 810)									
T1	<3.16	1.30	180/121	1.00		1.00		1.00	
T2	3.16–10.63	5.34	180/75	0.62	(0.44–0.88)	0.75	(0.50–1.13)	0.78	(0.51–1.20)
T3	≥10.63	24.63	180/74	0.61	(0.43–0.87)	0.84	(0.56–1.27)	0.78	(0.50–1.21)
*p* for trend ^a^				0.034		0.657		0.391	
Women (*n* = 435)									
T1	<6.14	2.50	96/63	1.00		1.00		1.00	
T2	6.14–22.08	11.35	97/42	0.66	(0.41–1.07)	0.80	(0.46–1.40)	0.83	(0.46–1.51)
T3	≥22.08	43.54	97/40	0.63	(0.39–1.02)	0.76	(0.44–1.34)	0.77	(0.42–1.39)
*p* for trend ^a^				0.117		0.421		0.440	
**Tomato**									
Total (*n* = 1245)									
T1	<4.70	1.70	276/217	1.00		1.00		1.00	
T2	4.70–20.12	10.15	277/120	0.55	(0.41–0.72)	0.66	(0.48–0.90)	0.66	(0.47–0.91)
T3	≥20.12	47.87	277/78	0.34	(0.25–0.47)	0.54	(0.38–0.77)	0.59	(0.41–0.85)
*p* for trend ^a^				<0.001		0.002		0.016	
Men (*n* = 810)									
T1	<4.03	1.33	180/144	1.00		1.00		1.00	
T2	4.03–16.68	8.75	180/72	0.50	(0.35–0.71)	0.67	(0.45–1.00)	0.64	(0.42–0.99)
T3	≥16.68	35.92	180/54	0.37	(0.26–0.54)	0.57	(0.37–0.88)	0.58	(0.37–0.92)
*p* for trend ^a^				<0.001		0.022		0.043	
Women (*n* = 435)									
T1	<6.13	2.74	96/67	1.00		1.00		1.00	
T2	6.13–30.90	14.03	97/54	0.79	(0.50–1.25)	0.98	(0.58–1.65)	0.97	(0.56–1.68)
T3	≥30.90	63.63	97/24	0.34	(0.20–0.60)	0.47	(0.25–0.87)	0.57	(0.30–1.12)
*p* for trend ^a^				<0.001		0.010		0.084	
**Tomato ketchup**									
Total (*n* = 1245)									
T1	<0.84	0.30	276/217	1.00		1.00		1.00	
T2	0.84–3.58	1.81	277/123	0.56	(0.42–0.74)	0.66	(0.49–0.90)	0.66	(0.48–0.92)
T3	≥3.58	8.34	277/75	0.33	(0.24–0.45)	0.51	(0.36–0.72)	0.55	(0.38–0.80)
*p* for trend ^a^				<0.001		0.001		0.005	
Men (*n* = 810)									
T1	<0.71	0.24	180/140	1.00		1.00		1.00	
T2	0.71–2.98	1.52	180/75	0.54	(0.38–0.76)	0.72	(0.49–1.08)	0.69	(0.45–1.06)
T3	≥2.98	6.45	180/55	0.39	(0.27–0.57)	0.61	(0.40–0.94)	0.62	(0.39–0.97)
*p* for trend ^a^				<0.001		0.039		0.067	
Women (*n* = 435)									
T1	<1.08	0.50	96/67	1.00		1.00		1.00	
T2	1.08–5.19	2.43	97/54	0.79	(0.50–1.25)	0.97	(0.58–1.64)	0.96	(0.56–1.67)
T3	≥5.19	10.94	97/24	0.34	(0.20–0.60)	0.47	(0.25–0.88)	0.58	(0.30–1.12)
*p* for trend ^a^				<0.001		0.011		0.089	

^a^ Adjusted for total energy intake using the residuals method. Model 1: Adjusted for age; Model 2: Adjusted for age, total caloric intake, family history of GC, smoking status, regular exercise, education level, occupation, and monthly household income; Model 3: Additionally adjusted for *H. pylori* infection. In the overall subjects, models 1, 2, and 3 were additionally adjusted for gender.

## Supplementary Materials

The following are available online at http://www.mdpi.com/2072-6643/10/8/1031/s1, Table S1: Gender-specific ORs and 95% CIs of GC according to tertiles of dietary lycopene intake stratified by *H. pylori* infection status, Table S2: Gender-specific ORs and 95% CIs of GC according to tertiles of dietary lycopene intake stratified by smoking status.
