# Supplementary material for: Dietary Carotenoids Intake and the Risk of Gastric Cancer: A Case—Control Study in Korea
Source: Nutrients. 2018 Aug 7;10(8):1031. doi: 10.3390/nu10081031 (PMC6115955; doi:10.3390/nu10081031)
Supplement: Supplementary file 1 [file nutrients-10-01031-s001.pdf]

**Table S1.** Gender-specific ORs and 95% CIs of GC according to tertiles of dietary lycopene intake stratified by *H. pylori* infection status [a].

| | Median Intake (μg/day) | *H. pylori*-Positive | | | | | *H. pylori*-Negative | | | | |
|---|---|---|---|---|---|---|---|---|---|---|---|
| | | No. of Controls/Cases | Model 1 | | Model 2 | | No. of Controls/Cases | Model 1 | | Model 2 | |
| | | | OR | (95% CI) | OR | (95% CI) | | OR | (95% CI) | OR | (95% CI) |
| **Lycopene** | | | | | | | | | | | |
| Men ($n$ = 790) | | | | | | | | | | | |
| T1 | 268.48 | 114/129 | 1.00 | | 1.00 | | 60/9 | 1.00 | | 1.00 | |
| T2 | 934.05 | 105/61 | 0.51 | (0.34–0.77) | 0.55 | (0.34–0.88) | 67/4 | 0.41 | (0.12–1.40) | 0.52 | (0.12–2.21) |
| T3 | 2, 963.59 | 114/62 | 0.48 | (0.32–0.72) | 0.57 | (0.36–0.91) | 60/5 | 0.54 | (0.17–1.70) | 0.86 | (0.21–3.45) |
| $p$ for trend [b] | | | 0.001 | | 0.043 | | | 0.417 | | 0.985 | |
| Women ($n$ = 431) | | | | | | | | | | | |
| T1 | 442.87 | 57/62 | 1.00 | | 1.00 | | 38/7 | 1.00 | | 1.00 | |
| T2 | 1,528.34 | 50/40 | 0.74 | (0.43–1.28) | 0.79 | (0.43–1.47) | 45/6 | 0.70 | (0.22–2.30) | 0.73 | (0.16–3.31) |
| T3 | 4, 843.59 | 46/28 | 0.56 | (0.31–1.02) | 0.68 | (0.35–1.33) | 50/2 | 0.22 | (0.04–1.10) | 0.30 | (0.04–2.03) |
| $p$ for trend [b] | | | 0.068 | | 0.282 | | | 0.064 | | 0.218 | |

[a] Dietary lycopene intake was categorized into tertiles according to the distribution of the control group among men (T1: <574.69, T2: 574.69–1,590.15, and T3: ≥1,590.15) and women (T1: <898.55, T2: 898.55–2,572.63, and T3: ≥2,572.63). [b] To test for trend across tertiles, the median intake for each tertile category was used as a continuous variable.

Model 1: adjusted for age; Model 2: adjusted for age, total caloric intake, family history of GC, smoking status, regular exercise, education level, occupation, and monthly household income.

**Table S2.** Gender-specific ORs and 95% CIs of GC according to tertiles of dietary lycopene intake stratified by smoking status [a].

| | Median Intake (μg/day) | Ever-Smoker [b] | | | | | Non-Smoker | | | | |
|---|---|---|---|---|---|---|---|---|---|---|---|
| | | No. of | Model 1 | | Model 2 | | No. of | Model 1 | | Model 2 | |
| | | Controls/Cases | OR | (95% CI) | OR | (95% CI) | Controls/Cases | OR | (95% CI) | OR | (95% CI) |
| **Lycopene** | | | | | | | | | | | |
| Men ($n$ = 810) | | | | | | | | | | | |
| T1 | 268.48 | 145/121 | 1.00 | | 1.00 | | 35/17 | 1.00 | | 1.00 | |
| T2 | 934.05 | 146/57 | 0.47 | (0.32–0.69) | 0.49 | (0.30–0.80) | 34/8 | 0.48 | (0.19–1.27) | 1.42 | (0.37–5.51) |
| T3 | 2,963.59 | 143/53 | 0.44 | (0.30–0.66) | 0.45 | (0.27–0.73) | 37/14 | 0.78 | (0.33–1.82) | 1.44 | (0.43-4.79) |
| *p* for trend [c] | | | <0.001 | | 0.005 | | | 0.816 | | 0.628 | |
| Women ($n$ = 434) | | | | | | | | | | | |
| T1 | 442.87 | 2/5 | 1.00 | | 1.00 | | 94/63 | 1.00 | | 1.00 | |
| T2 | 1,528.34 | 4/7 | 0.50 | (0.05–4.67) | - | - | 93/39 | 0.63 | (0.38–1.02) | 0.78 | (0.44–1.38) |
| T3 | 4,843.59 | 6/4 | 0.20 | (0.02–1.86) | - | - | 91/26 | 0.43 | (0.25–0.73) | 0.64 | (0.34–1.20) |
| *p* for trend [c] | | | 0.139 | | - | | | 0.003 | | 0.183 | |

[a] Dietary lycopene intake was categorized into tertiles according to the distribution of the control group among men (T1: <574.69, T2: 574.69–1,590.15, and T3: ≥1,590.15) and women (T1: <898.55, T2: 898.55–2,572.63, and T3: ≥2,572.63). [b] Subjects who currently smoke or previously smoked were combined as ever-smokers. [c] To test for trend across tertiles, the median intake for each tertile category was used as a continuous variable.

Model 1: adjusted for age; Model 2: adjusted for age, total caloric intake, family history of GC, smoking status, regular exercise, education level, occupation, and monthly household income.
